# Association of COVID-19 with diabetes: a systematic review and meta-analysis

**DOI:** 10.1038/s41598-022-24185-7

**Published:** 2022-11-23

**Authors:** Paddy Ssentongo, Yue Zhang, Lisa Witmer, Vernon M. Chinchilli, Djibril M. Ba

**Affiliations:** 1 Department of Public Health Sciences, Penn State College of Medicine, Hershey, PA USA; 2 Department of Medicine, Penn State Health Medical Center, Hershey, PA USA; 3 Department of Public Health Sciences, Penn State College of Medicine, 90 Hope Drive, Suite 2200|MC A210, Hershey, PA 17033 USA

**Keywords:** Medical research, Infectious diseases, Viral infection

## Abstract

Emerging evidence suggests that coronavirus disease-2019 (COVID-19) may lead to a wide range of post-acute sequelae outcomes, including new onset of diabetes. The aim of this meta-analysis was to estimate the incidence of newly diagnosed diabetes in survivors of COVID-19. We searched MEDLINE, Scopus, Cochrane Central Register of Controlled Trials and the World Health Organization Global Literature on Coronavirus Disease and clinical trial registries for studies reporting the association of COVID-19 and diabetes. Search dates were December 2019–October 16, 2022. Two investigators independently assessed studies for inclusion. Risk of bias was assessed using the Newcastle–Ottawa Scale. We estimated the effect of COVID-19 on incident diabetes by random-effects meta-analyses using the generic inverse variance method. We identified 8 eligible studies consisting of 4,270,747 COVID-19 patients and 43,203,759 controls. Median age was 43 years (interquartile range, IQR 35–49), and 50% were female. COVID-19 was associated with a 66% higher risk of incident diabetes (risk ratio, 1.66; 95% CI 1.38; 2.00). The risk was not modified by age, sex, or study quality. The median risk of bias assessment was 7. In this systematic review and meta-analysis, COVID-19 was associated with higher risk for developing new onset diabetes among survivors. Active monitoring of glucose dysregulation after recovery from severe acute respiratory syndrome coronavirus 2 (SARS-CoV-2) infection is warranted.

## Introduction

The severe acute respiratory syndrome coronavirus 2 (SARS-CoV-2), the causative strain of coronavirus disease 2019 (COVID-19) was first detected in early December 2019 in Wuhan, China. As of October 16, 2022, more than 625 million COVID-19 cases and 6.6 million deaths were reported globally^1^.

Post-COVID or long COVID-19 conditions are a wide range of new, returning, or ongoing health problems that individuals experience after first being infected with the virus that causes COVID-19^2^. Emerging evidence suggests that COVID-19 may lead to a wide range of post-acute sequelae outcomes, including new onset of diabetes^3–8^. The exact mechanisms for incident diabetes in survivors of COVID-19 are not well understood, but it is likely that complex interrelated processes are involved, including previous stress hyperglycemia, steroid-induced hyperglycemia, and direct or indirect effects of SARS-CoV-2 on the β-cells of pancreatic islets^4,6,7^.

A previous study with more than 180,000 veterans found that patients who survived COVID-19 were 40% more likely to develop diabetes than those who were never diagnosed with COVID-19^9^. Moreover, another study found that up to 14% of people hospitalized for COVID-19 were diagnosed with diabetes later^10^. However, to date, there is no study that has systematically synthesized the available evidence for the association of COVID-19 with new onset diabetes. A previous systematic review and meta-analysis was limited to only a proportion of newly diagnosed diabetes after COVID-19 with no comparison groups^10^. We aim to fill this critical knowledge gap by conducting a systematic review and meta-analysis to determine the association of COVID-19 with incident diabetes.

## Methods

This study is being reported following Preferred Reporting Items for Systematic Reviews and Meta-analyses (PRISMA) 2020^11^. This study was deemed exempt by the Penn State Institutional Review Board.

### Data sources and searches

We searched MEDLINE, Scopus, Cochrane Central Register of Controlled Trials and the World Health Organization Global Literature on Coronavirus Disease and clinical trial registries for studies reporting the association of COVID-19 and diabetes without language restriction. Search dates were December 2019–October 16, 2022. The following Medical Subject Headings and keyword search terms were used; [“diabetes” OR type 2 diabetes OR type 1 diabetes OR “type 1 diabetes mellitus” OR “type 2 diabetes mellitus OR “diabetes mellitus”] AND [“SARS-CoV-2” OR “COVID-19” OR “severe acute respiratory syndrome coronavirus-2” OR “coronavirus disease 2019”].

### Study selection

Participant (P) Exposure (E) Comparator [C], Outcome (O) Study type (S) [PECOS] criteria was used to select studies^12^:

*Participants* Persons of all ages and sex included in studies that investigated incident diabetes in survivors of COVID-19.

*Exposure* COVID-19.

*Comparison* Non-COVID-19 group.

*Outcome of interest* Diabetes.

*Study type* Observational studies.

Pairs of independent investigators (YZ and DMB) screened the titles and abstracts of all citations and screened the full-text version of eligible studies. Disagreements in the included papers were resolved by discussion and if necessary, a third investigator (PS) was consulted.

### Data extraction and quality assessment

Two investigators (YZ and DMB) worked independently to extract study the following date: authors, publication year, country of the study, study design, study-level descriptive statistics (mean (SD)/median (IQR) age in years, proportion (%) female), sample size, number with diabetes, number with COVID-19, outcome assessment, follow-up time, number of controls, risk ratio and 95% confidence interval. Newcastle–Ottawa Scale for observational studies was used to evaluate the risk of bias^13^. Studies with fewer than 5 stars were considered low quality; 5 to 7 stars, moderate quality; and more than 7 stars, high quality.

### Data synthesis and analysis

The primary outcome was incident diabetes in survivors of COVID-19. For studies without measures of associations, a generalized linear mixed model was used to calculate the RR using the number of events and the sample size of each study group^14^. One study Barret et al. (2022) used two different national databases and reported separate results. Therefore, in this circumstance, we separated the effect estimates from Barret et al. study into two studies as one with IQVIA database and the second one with HealthVerity^3^. A study by McKeigure and colleagues reported two separate RRs for diabetes associated with COVID-19 at various time points, therefore, a fixed-effects model was utilized to pool the estimate within the study before conducting the random-effect meta-analysis. The pooled RR estimate for diabetes risk from each study was weighted by the inverse of its variance (inter-study plus intra-study variances). Pooled inter-study variance (heterogeneity) was estimated by DerSimonian and Laird (DL) random-effects method^15^. Heterogeneity between studies was evaluated with the ${I}^{2}$ indicator expressed as percent low (25%), moderate (50%), and high (75%)^16^. Egger’s linear regression and Begg’s rank tests were employed to quantitatively evaluate publication bias^17,18^ and qualitatively with funnel plots. Statistical significancy was set at p < 0.05. All statistical analyses were performed with R software version 3.6.2 (R Foundation for Statistical Computing, Vienna, Austria) using Meta and *Metafor* R packages.

## Results

### Identified studies

**Figure 1:**
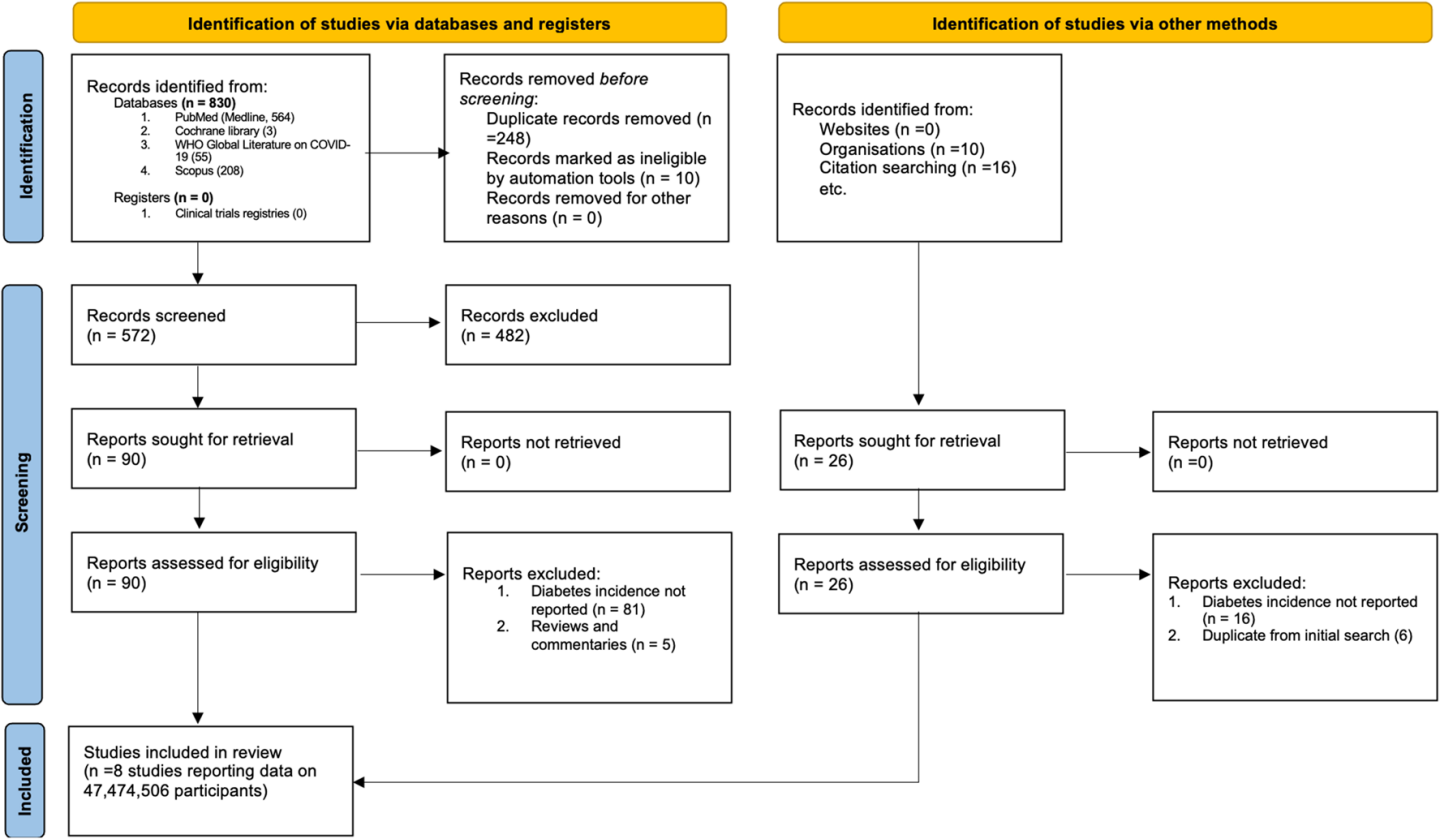
PRISMA flow chart of a systematic review of diabetes incidence in survivors of COVID-19. PRISMA, Preferred Reporting Items for Systematic Reviews and Meta-Analyses.

Figure 1 summarizes study selection process. A total of 853 studies were screened. The exclusion process yielded 8 studies^3,5,9,19–23^ conducted in 3 countries. Barret et al. was reported in this meta-analysis as two independent studies^3^. The baseline characteristics of the studies included in the systematic review are presented in Table 1. Included studies consisted of patients 47,474,506 participants, with median age of 43 years (IQR 35–49), and 50% were female. The median study quality was 7 (range 5–9).

**Table 1:** Meta-analysis characteristics of included cohort studies reporting COVID-19 and risk of diabetes.

Author (year)	Sample size, N	Female, N (%)	Outcome (diabetes) assessment	Country	Study design	Mean age (y)	Total Case, N	Follow-up periods	Median follow-up time (D)	Reported effect sizes: HR/RR (95% CI)	Covariates in the fully-adjusted model	Quality score	COVID-19 patients	Controls
Rathmann et al. (2022)	71,730 (35,865 pairs)	32,732 (45.6%)	ICD-10 codes (E11-E14)	Germany	Retrospective cohort study	42.6	364	March 2020 to January 2021	119	IRR: 1.28 (1.05, 1.57)	Sex, age, health insurance, index month for Covid-19 and comorbidity (obesity, hypertension, hyperlipidaemia, myocardial infarction, stroke)	8	35,865	35,865
Barret et al. (2022)	485,358	243,102 (50.1%)	ICD-10 codes (E08-E13)	US	Retrospective cohort study	12.3	200	March 2020 to February 2021	NA	HR: 2.66 (1.98, 3.56)	Matched on age, sex, and month of encounter	7	80,893	404,465
Barret et al. (2022)	878,878	440,024 (50.1%)	ICD-10 codes (E08-E13)	US	Retrospective cohort study	12.7	1973	March 2020 to June 2021	NA	HR: 1.31 (1.20, 1.44)	Age, sex, and month of encounter	7	439,439	439,439
Xie et al. (2022)	4,299,721	485,021 (11.3%)	ICD-10 codes (E08.X to E13.X) or a HbA1c measurement of more than 6·4% (46 mmol/mol)	US	Cohort study	60.9	134,873	March 2020 to Sept 2021	352	HR: 1.40 (1.36, 1.44)	Age, race, sex, area deprivation index, BMI, smoking status, use of long-term care, number of outpatient and inpatient encounters, and number of HbA1c measurements; comorbidities including cancer, cardiovascular disease, cerebrovascular disease, chronic lung disease, dementia, HIV, hyperlipidaemia, and peripheral artery disease; laboratory test results including estimated glomerular filtration rate (eGFR) and HbA1c; vital signs including systolic and diastolic blood pressure; and medications including the use of steroids	9	181,280	4,118,441
Wander et al. (2022)	2,777,768	376,274 (13.5%)	(1) two or more abnormal laboratory values from plasma or serum (random glucose ≥ 200 mg/dL, fasting glucose ≥ 126 mg/dL, 2-h glucose from an oral glucose tolerance test ≥ 200 mg/dL) or whole blood (A1C ≥ 6.5%); or (2) two outpatient or one inpatient ICD-10 codes of E08–E13; or (3) receipt of an initial and one refill prescription of a glucose-lowering medication	US	Retrospective cohort study	59	9150	March 2020 to March 2021	120	OR for male: 2.56 (2.32, 2.83) OR for female: 1.21 (0.88, 1.68)	Age, race, ethnicity, BMI, tobacco use, and facility location	9	126,710	2,651,058
Daugherty et al. (2021)	9,247,505	4,607,112 (49.8%)	ICD-10 codes	US	Retrospective cohort study	42.4	1886	January 2020 to October 2020	95	HR: 2.47 (1.14, 5.37)	Propensity score matching with age, sex, socioeconomic status, race, index month, pre-existing comorbidities, total length of stay as an inpatient in the previous year, previous number of visits to a primary care physician, cardiologist, or nephrologist	5	266,586	8,980,919
Qeadan et al. (2022)	27,292,879	13,755,616 (54.1%)	ICD-10 codes	US	Retrospective cohort study	45.4	5163	December 2019 to July 2021	NA	OR: 1.42 (1.38, 1.46)	Age, gender, race and ethnicity, marital status, and US geographical region	6	2,489,266	24,803,613
Kendall et al. (2022)	571,256 (285,628 matched pairs)	142,288 (49.8%)	NA	US	Matched Retrospective cohort study	9.3	123	2020 to 2021	NA	HR: 1.83 (1.36, 2.44)	Propensity score matching with age, sex, race, ethnicity, family history of diabetes	9	285,628	285,628
McKeigure et al. (2022)	1,849,411	924,706 (50%)	ICD-10 codes (E10–E14) or an outpatient consultation with specialty coded A81 for diabetes	UK	Retrospective cohort study	NA	1074	March 2020 to November 2021	NA	"RR: 0.86 (0.62, 1.21) for infection > 30 days RR: 2.62 (1.81, 3.78) for infection within 30 days	Age, sex, and number of vaccine doses at least 14 days before	7	365,080	1,484,331

### Association of COVID-19 and incident diabetes

**Figure 2:**
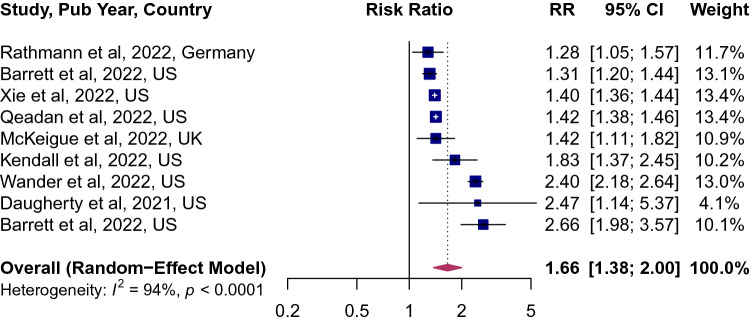
Forest plot for the overall pooled estimate for the association of COVID-19 and incident diabetes. Effect size values represent risk ratio and corresponding 95% CI. Blue squares and their corresponding lines are the point estimates of each study and 95% confidence intervals (95% CI). Maroon diamonds represent the pooled estimate (width denotes 95% CI). Heterogeneity (*I*^2^ = 94%, p for heterogeneity < 0.0001; 8 studies).

**Figure 3:**
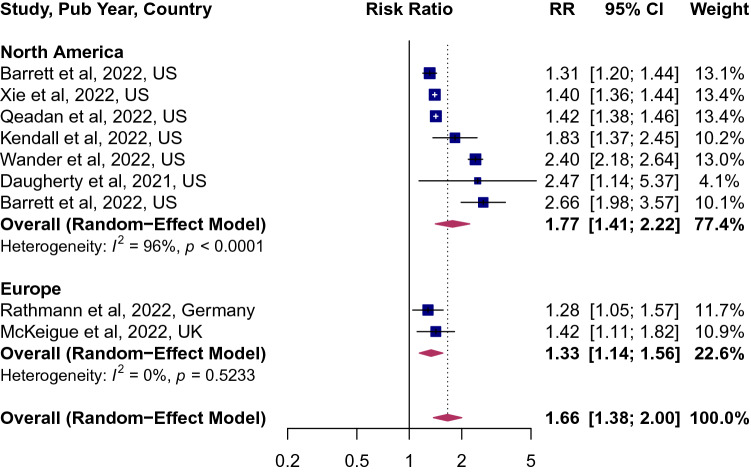
Forest plot of studies stratified by geographic regions.

Of the 8 studies that characterized the risk of incident diabetes among survivors of COVID-19, the pooled point estimates was 1.66 (95% CI 1.38; 2.00, Fig. 2), implying a 66% higher risk of diabetes. The between-study variation was high (*I*^2^ = 94, p < 0.0001). The risk was not modified by age, sex and study quality (Supplemental Table 1). However, when studies were stratified by geographic region, the risk was higher in studies from the United States 1.77 (95% CI 1.41; 2.22, Fig. 3), compared to those in Europe 1.33 (95% CI 1.14; 1.56).

### Publication bias and study heterogeneity

**Figure 4:**
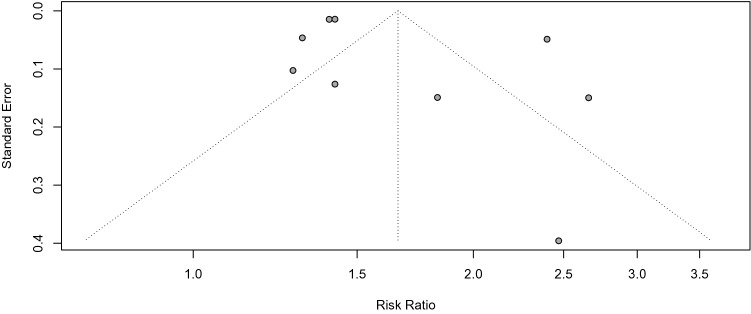
Funnel plots to assess potential for small-study publication bias^26^. Symmetrical inverted funnel plot suggested absence of publication bias.

**Figure 5:**
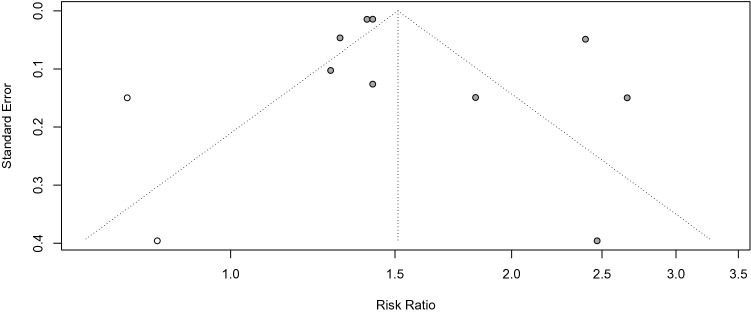
Funnel plots from trim and fill analysis. Duval & Tweedie trim and fill analytical method suggests that the adjusted effect estimates would fall in the range of 1.21 to 1.88, and 2 studies were added^24^.

**Figure 6:**
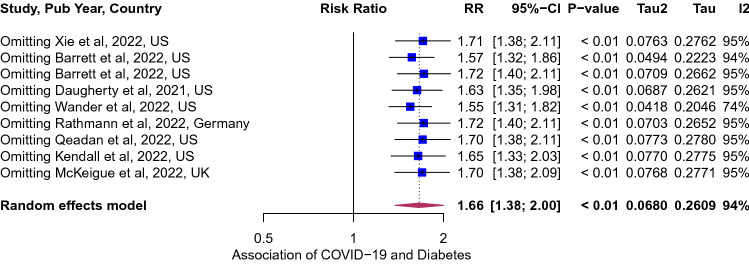
Influence and outlier (leave-one-out meta-analysis) analysis for the association of COVID-19 and incident diabetes^27^. The results of our outlier and influence analysis show the recalculated pooled point estimate ranged from 1.55 to 1.72 when one study was omitted each time.

Funnel plot of the included studies (Fig. 4) indicated asymmetry suggesting lack of publication bias. Quantitative analysis of publication bias with Egger’s test (p = 0.053) and Begg's test (p = 0.06) were non-significant. Duval and Tweedie's trim and fill test was conducted to balance the funnel plots and adjust for potential publication bias^24^. The analysis showed that if publication bias existed, 2 additional studies will be needed to eliminate bias and the overall effect of COVID-19 on incident diabetes changed from 1.66 (95% CI 1.38; 2.00 to 1.51 (1.21; 1.88, Fig. 5). Next, we performed influence sensitivity analyses by excluding and replacing one study at a time from the meta-analysis and calculated the RR for the remaining studies^25^. No substantial change from any of the pooled RR was observed when other studies were removed in turn, indicating that no individual study had a considerable influence on the pooled estimate. The plots for the analysis estimates are provided in Fig. 6.

## Discussion

### Principal findings

In this systematic review and meta-analysis of 8 cohort studies including over 47 million participants, COVID-19 was associated with a 66% higher risk of diabetes compared to the controls without COVID-19. The risk was not modified by age, sex, and study quality. The risk of bias assessment was low.

Our findings are consistent with the previous meta-analysis that assessed the proportion of COVID-19 survivors with incident diabetes. A 2021 study by Sathish and colleagues assessed a total of 3711 COVID-19 patients with 492 cases of newly diagnosed diabetes from eight studies^10^. In the random-effects meta-analysis model, the estimated pooled proportion of incident diabetes was 14.4% (95% CI 5.9–25.8%). They, however, noted a high degree of heterogeneity (*I*^2^ 98.6%, p < 0.001). The weaknesses of the above study, however, included a lack of a control group and a very small study sample size.

Potential pathophysiological mechanisms of new-onset diabetes among COVID-19 survivors are complex and not fully understood. SARS-CoV-2 binds to angiotensin-converting enzyme 2 and transmembrane serine protease 2 receptors, which are expressed in key metabolic organs and tissues, including pancreatic beta cells, adipose tissue, the small intestine, and the kidneys^28–30^. Furthermore, it has been demonstrated that SARS-CoV-2 infection attenuates pancreatic insulin levels and secretion and induces β cell apoptosis^31,32^. Thus, it is plausible that SARS-CoV-2 may cause pleiotropic alterations of glucose metabolism that could lead to incident diabetes or facilitate a rapid transition from the prediabetes state to full-blown diabetes. SARS-CoV-2 is not the only virus associated with diabetes. A significant number of other viruses are associated with type 1 diabetes through molecular mimicry, including Coxsackievirus B, rotavirus, mumps virus, and cytomegalovirus^33–35^. Furthermore, findings from prospective studies have demonstrated a temporal association between hepatitis C virus and type 2 diabetes^36^.

### Clinical implications of our findings and recommendations

Given the extraordinary number of COVID-19 survivors globally, the modest increase in diabetes risk could correspond to a drastic rise in the number of people diagnosed with the disease worldwide. Therefore, active monitoring of glucose dysregulation after recovery from severe COVID-19 infection is warranted. Additionally, there is a need for studies that determine various social determinants of health associated with new onset diabetes. These factors would be critical to developing effective prevention and management strategies for the disease. Lastly, future research could also focus on employing genomics data to stratify acute COVID-19 patients and predict phenotypes of patients at an increased risk of COVID-19- induced diabetes and uncover novel disease mechanisms.

### Limitations

Our study has some limitations worth noting. First, a high degree of heterogeneity was observed, which could have been caused by pooling studies from different sociodemographic populations. Nevertheless, a random effects model was invoked to derive plausible estimates. Second, it is also a possibility that some individuals in the control groups could have had undetected mild or asymptomatic COVID-19 because they had not been tested. Such non-differential misclassification of the exposure may underestimate the strength of the association of COVID-19 with the onset of diabetes. Lastly, due to the limited number of studies included in the present meta-analysis, we did not categorize the risk by the type of diabetes such as type 1 and type 2.

### Conclusions

In this systematic review and meta-analysis, COVID-19 was a risk factor for developing new onset diabetes among survivors. Active monitoring of glucose dysregulation after recovery from severe acute respiratory syndrome coronavirus 2 infection is warranted.

## Supplementary Information

Supplementary Table 1.

Supplementary Information 2.

## Data Availability

All data generated for this study are included in this manuscript.
