# Supplementary material for: Association of COVID-19 with diabetes: a systematic review and meta-analysis
Source: Sci Rep. 2022 Nov 23;12:20191. doi: 10.1038/s41598-022-24185-7 (PMC9684130; doi:10.1038/s41598-022-24185-7)
Supplement: Supplementary file 1 — Supplementary Table 1. [file 41598_2022_24185_MOESM1_ESM.docx]

Supplemental Table 1: Meta-regression results for the association between COVID-19 and diabetes

| **Variable** | **N studies** | **Coefficient 95% CI** | **p-value** | **R^2^** |
| --- | --- | --- | --- | --- |
| Sex (Per 5% increase in female prop | 8 | -0.01 (-0.076, 0.055) | 0.7491 | 0 |
| Age (Per 5-year increase in mean age) | 8 | -0.003 (-0.067, 0.061) | 0.9296 | 0 |
| Study quality | 9 | -0.02(-0.20, 0.161) | 0.835 | 0 |
